# Few-shot prototype adaptation for generalizable electromyography gesture recognition

**DOI:** 10.1038/s41598-026-40352-6

**Published:** 2026-03-07

**Authors:** Hunmin Lee, Brian Lim, Ming Jiang, Zhi Yang, Qi Zhao

**Affiliations:** 1 Department of Computer Science, University of Minnesota, Minneapolis, 55455 USA; 2 Department of Biomedical Engineering, University of Minnesota, Minneapolis, 55455 USA

**Keywords:** Electromyography, Few-shot learning, Gesture recognition, Prototype learning, Human-computer interaction, Prosthetic control, Engineering, Mathematics and computing

## Abstract

We present EMG-Adapt, a novel few-shot prototype adaptation framework designed to enhance the robustness and data efficiency of electromyography (EMG)-based gesture recognition. By integrating the representational power of prototype learning with the rapid adaptation capabilities of meta-learning, our framework introduces several technical novelties. These include a cepstrum coefficient average feature extraction method that reduces sensitivity to noise and variations, a deep prototype learning method based on hybrid loss functions for both discriminative classification and embedding space structure, and a meta-learning strategy for efficient prototype update with minimal labeled examples. Our integrated approach significantly improves few-shot gesture recognition performance, requiring substantially less calibration data than conventional methods. Extensive experiments on five public EMG datasets demonstrate state-of-the-art performance in cross-session and cross-user generalization scenarios, while maintaining computational efficiency. This work represents a significant advancement towards practical, user-friendly, and scalable EMG-based human-computer interfaces, with potential applications in prosthetics, assistive technologies, and virtual reality. Future research will explore self-supervised learning techniques and extend the framework to handle more gestures and online adaptation strategies for enhanced real-world robustness.

## Introduction

Electromyography (EMG)-based gesture recognition has emerged as a critical technology for restoring motor function in individuals with upper-limb impairments, offering life-changing improvements in mobility and independence ^1^. While EMG signals provide rich neuromuscular information, their non-stationary nature and subject-specific variability create fundamental barriers to reliable clinical deployment ^2,3^. Despite remarkable progress in deep learning for biosignal processing ^4^, current systems remain constrained by their inability to tackle the variability of EMG signals across time and different users, particularly in the context of clinical applications where data collection is severely limited due to patient availability, muscle fatigue, and the impracticality of extensive data recording sessions.

The generalization challenges in EMG-based gesture recognition can be characterized across different dimensions. First, longitudinal generalization poses unique challenges as EMG signals exhibit significant drift over time. This variability stems from factors such as electrode placement inconsistencies, muscle fatigue, and variations in environmental conditions, making it difficult to maintain consistent performance over time ^5,6^. Second, cross-user generalization remains a significant hurdle, as EMG signals vary substantially across individuals due to differences in muscle anatomy, subcutaneous fat distribution, and idiosyncratic movement patterns ^7,8^. Such physiological and behavioral variations often cause models trained on one user’s data to perform poorly when applied to new users ^8,9^. These combined challenges create a complex generalization landscape that demands innovative solutions beyond conventional deep learning approaches.

To address these challenges, we propose EMG-Adapt, a novel few-shot prototype adaptation framework for generalizable and adaptive EMG-based gesture recognition. Our framework uniquely integrates feature engineering, model architecture innovations, and domain adaptation strategies while maintaining robust performance under data-limited conditions. Unlike prior studies that typically focus on individual aspects in isolation, our holistic approach combines these complementary strategies into a unified framework that achieves superior generalization. The main contributions of this work are:

- A novel cepstrum coefficient average (CCA) feature extraction, designed to support cross-session generalization by capturing stable spectral patterns in EMG signals while reducing sensitivity to transient noise and variations;
- A deep prototype learning architecture to address cross-user generalization by learning stable and separable gesture prototypes with a hybrid objective combining prototype and cross-entropy losses;
- A few-shot adaptation strategy, effective in both cross-session and cross-user settings, which enables rapid model adaptation using minimal training data by dynamically updating prototypes via meta-learning;
- Extensive experiments on multiple public EMG datasets, demonstrating state-of-the-art performance in both cross-session and cross-user recognition tasks, particularly in few-shot scenarios.

The remainder of this paper is organized as follows: the Related Works section reviews prior work in EMG-based gesture recognition and few-shot learning. The Methodology section details our proposed framework and its components. The Experiments section presents experimental results and analysis. Finally, the Conclusion section discusses implications and future directions.

## Related works

### DNN approaches to EMG-based gesture recognition

Deep Neural Networks (DNNs) have transformed EMG-based gesture classification by surpassing traditional machine learning methods. Their key advantage lies in automatically extracting meaningful features from raw EMG signals, eliminating the need for manual feature engineering ^10^. Various DNN architectures have demonstrated exceptional capabilities in modeling complex EMG patterns, including Convolutional Neural Networks (CNNs) ^11–24^, Recurrent Neural Networks (RNNs)-based variants ^14,25–28^, Graph Neural Networks (GNNs) ^29,30^, and hybrid approaches ^14,19,27,28^.

The evolution of DNN architectures in EMG processing has followed two main trajectories. The first approach leverages 2D-CNNs by transforming EMG signals into image-like representations ^11–18,20–24^. Notable examples include AtzoriNet ^11^, which pioneered the application of 2D-CNNs to EMG images, and subsequent improvements like GengNet ^12^ and DuNet ^13^. Hybrid architectures such as HuNet ^14^, MVCNN ^15^, and CMAM ^27^ enhanced performance by combining 2D-CNNs with LSTMs to capture both spatial and temporal dynamics. Recent advances like sEMGXCM ^18^ and EMGHandNet ^19^ have further refined this hybrid approach. The second trajectory explores 1D-CNNs, which process EMG signals in their natural sequential form. Models such as XceptionTime ^31^ and XCM ^32^ demonstrate that 1D-CNNs can achieve comparable accuracy while requiring less computational resources, making them particularly suitable for real-time applications.

While these DNN approaches have achieved remarkable performance in well-controlled experiments, they face significant challenges in generalization. Benchmarking studies ^33–38^ consistently report a substantial performance gap when models are applied to unseen individuals, largely due to physiological variability, electrode placement inconsistency, and environmental factors. Although prosthetic systems are ultimately personalized, achieving cross-subject generalization remains crucial for practical usability. A model that generalizes across users can substantially reduce the calibration effort required for a new amputee, enabling faster adaptation and reducing the dependence on extensive subject-specific data. This capability is particularly beneficial for initializing models prior to personalization, for population-level pretraining, and for developing transferable prosthetic control strategies ^38^. Our work specifically addresses these limitations by introducing novel architectural and learning mechanisms that explicitly target model generalization and few-shot adaptation, distinguishing our approach from previous solutions that primarily focused on improving single-user performance ^11–14,16,18,24,39,40^.

### Addressing generalization challenges

The primary challenge in EMG-based gesture recognition lies in achieving robust performance across diverse users and conditions. Researchers have explored various strategies to enhance model generalization. Recent advances suggest different domain adaptation techniques ^25^, including iterative self-training approaches that refine pseudo-labels to enhance target-domain recognition ^41^, self-adaptive dimensional distribution adaptation techniques that automatically select optimal feature domains for cross-individual alignment ^42^, and shallow UDA frameworks that leverage Linear Discriminant Analysis with K-Means to improve cross-subject performance without explicit calibration ^43^. Similarly, transfer learning methods aim to leverage knowledge from source domains to improve target domain performance ^17,44^. However, these approaches typically require substantial data from both domains, limiting their practicality when dealing with new users or changing conditions.

To address the data scarcity challenge, recent research has shifted towards meta-learning and few-shot learning approaches ^20,45–48^. Meta-learning, or “learning to learn”, trains models to rapidly adapt to new tasks by leveraging knowledge from related tasks, while few-shot learning enables a model to recognize new classes using only a few labeled examples, mimicking human learning capabilities ^49^. Notable contributions include FS-HGR ^45^, which pioneered few-shot gesture recognition using temporal convolutions, and CSAC-Net ^46^, which combined model-agnostic meta-learning with attention mechanisms to improve adaptability. Building on these models, recent works explored metric-based approaches such as Siamese networks ^50^, which learn to classify gestures by comparing input similarities. A particularly promising direction is prototype learning, where each class is represented by a learnable prototype vector in an embedding space. This structure supports distance-based classification and performs well in low-data scenarios ^20,37,47,48^. However, most prototype-based methods rely on static representations, limiting their ability to adapt to user variability.

Our work distinguishes itself by bridging the gap between static prototype learning and adaptive meta-learning. We propose a unified framework that combines a dynamic prototype refinement mechanism with a meta-learning strategy for efficient few-shot adaptation. This integrated approach enables three key advances: (1) it improves cross-session and cross-user generalization by dynamically refining prototypes to reflect distributional variations observed in new data based on minimal samples; (2) it increases contextual flexibility by modulating prototype representations in a task-specific manner, enabling the model to better capture the structural nuances of each learning scenario; and (3) it achieves rapid and data-efficient adaptation without sacrificing accuracy. By addressing multiple dimensions of generalization in a single framework, our method provides a novel and scalable solution beyond what prior domain adaptation, meta-learning, or prototype learning approaches can achieve independently.

## Methodology

Gesture recognition systems based on EMG signals face significant challenges due to signal variability. These signals naturally vary across subjects, sessions, and recording conditions due to individual physiological differences, electrode placement changes, muscle fatigue, and environmental noise. Traditional machine learning approaches struggle with these variations, often requiring extensive retraining when applied to new subjects or conditions. Moreover, the collection of comprehensive labeled EMG datasets is impractical, as the process is both time-consuming and potentially fatiguing for participants.

**Fig. 1:**
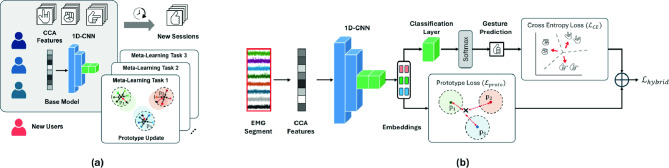
(**a**) High-level pipeline of the proposed framework, illustrating the stages from raw EMG signal input to gesture classification. (**b**) Detailed overview of the feature extraction and prototype learning approach. Raw EMG signals are first processed with CCA-based feature extraction and then passed through a specialized 1D-CNN architecture to generate embeddings. In the architecture, blue blocks denote 1D convolutional layers and green blocks denote fully connected layers. The model is optimized using a hybrid loss that combines cross-entropy with prototype-based metrics.

Our EMG-Adapt framework addresses these challenges by integrating feature engineering, prototype learning, and meta-learning approaches. This integration is guided by two fundamental insights: EMG signals from identical gestures share underlying patterns that can be captured through prototypical representations, and effective generalization depends on both robust feature extraction and efficient adaptation mechanisms. An overview of the proposed framework is visualized in Fig. 1. Our framework introduces three key technical innovations:

- A novel feature extraction method based on averaged cepstrum coefficients, designed to capture stable and discriminative spectral features in highly variable EMG signals. By reducing sensitivity to transient noise and signal variability by averaging cepstral coefficients across frequency bins, it is uniquely effective for cross-session generalization, contributing to reliable gesture recognition under limited data conditions.
- A specialized 1D-CNN-based prototype learning architecture that develops discriminative gesture representations through explicit prototype modeling. The system employs a hybrid loss function combining cross-entropy with prototype-based similarity metrics, enabling simultaneous optimization of classification accuracy and the learning of stable, geometrically-structured representations;
- An adaptive few-shot learning approach that incorporates meta-learning principles for efficient model adaptation. By combining a dynamic prototype update mechanism with the Reptile algorithm, this strategy enables rapid model personalization using minimal training data while maintaining robust recognition performance through controlled prototype evolution;

The following subsections outline the key components of our framework: feature extraction, prototype learning with a hybrid loss, and meta-learning for adaptive prototype updates. Together, these elements support effective cross-user and cross-session gesture recognition with minimal adaptation data.

### Feature extraction for EMG signal representation

EMG-based gesture recognition is challenged by signal variability across subjects and sessions. Many existing feature extraction methods often lack generalizability due to sensitivity to noise and subject-specific differences ^51–56^. We address this by averaging cepstrum coefficients, which capture consistent spectral patterns while smoothing transient variations. Unlike traditional approaches, our method computes averaged cepstrum coefficients across both time and channels, which suppresses transient noise and highlights the spectral components that are consistently associated with each gesture. This aggregation distills the essential characteristics of the EMG signal by emphasizing patterns that are stable across repetitions and less sensitive to subject- or session-specific variations. As a result, CCA provides a compact, informative representation that enhances robustness, cross-session generalization, and few-shot learning performance.

Our pipeline begins by preprocessing raw multi-channel EMG signals to improve quality. A first-order Butterworth low-pass filter with a 1 Hz cutoff frequency was applied bidirectionally (zero-phase), thereby removing motion artifacts and baseline drift without introducing phase distortion, consistent with prior work ^12,22,55,57^. Filtered signals are then segmented using a sliding window ^55^. For each segment, we compute cepstrum coefficients in three steps:

First, we transform the preprocessed time-domain signal *x*(*t*) into the frequency domain using Fourier Transform $\mathcal {F}(\cdot )$ and compute its power spectrum:

1 $$\begin{aligned} P(k) = |\mathcal {F}\left( x(t)\right) |^2 \end{aligned}$$

where *k* is the frequency index, and *P*(*k*) represents the power at frequency *k*.

Next, we apply a logarithmic transformation to the power spectrum to compress its dynamic range and highlight subtle spectral variations. The cepstrum coefficients are then obtained through an Inverse Fourier Transform $\mathcal {F}^{-1}(\cdot )$ :

2 $$\begin{aligned} q(n) = \mathcal {F}^{-1} \left( \log P(k) \right) \end{aligned}$$

where *q*(*n*) denotes the cepstral coefficient at quefrency index *n* ^58^ (Cepstral Coefficients Section in the Supplementary Information).

Finally, we compute the average of these coefficients across all quefrency indices:

3 $$\begin{aligned} \bar{q} = \frac{1}{N} \sum _{n=1}^{N} q(n) \end{aligned}$$

This averaging operation is crucial as it captures dominant spectral characteristics while reducing sensitivity to noise by smoothing out variations in signal amplitude. For each electrode channel *e*, $\bar{q}_e$ is computed, and concatenated to form the model input vector:

4 $$\begin{aligned} \textbf{x}_i = [\bar{q}_{e_1}, \bar{q}_{e_2}, ..., \bar{q}_{e_E}] \end{aligned}$$

where *E* is the number of electrode channels and $\textbf{x}_i$ represents the feature vector corresponding to the *i*-th sample. This representation demonstrates superior robustness compared to traditional spectral features (detailed in Ablation Studies - Feature Selection Section), making it especially effective for few-shot learning scenarios where limited training data is available. Further details on the computation and properties of cepstral coefficients are provided in the Supplementary Information (Cepstral Coefficients Section).

### Deep prototype learning for robust gesture classification

Conventional classifiers often struggle with EMG gesture recognition due to their implicit class representations, leading to poor generalization ^29,33,59,60^. We propose a prototype-guided learning approach that explicitly models each gesture class as a prototypical embedding, serving as an anchor during training. This promotes intra-class compactness and inter-class separation, improving robustness to subject/session variability and enabling better generalization, especially in few-shot scenarios.

Our embedding function $f_{\theta }(\cdot )$ is implemented via a specialized 1D-Convolutional Neural Network (1D-CNN), which efficiently processes sequential EMG features in their native 1D form. Unlike conventional 2D-CNNs ^15,17,19,21–23,27–29,45,55^ or handcrafted features ^51–56^, this architecture balances accuracy and computational cost. As detailed in Supplementary Table S1 and Supplementary Fig. S3, the network includes four convolutional blocks (with batch normalization and ReLU activation), followed by flattening and three fully-connected layers. This structure maps input features into a 128-dimensional embedding space optimized for gesture discrimination.

Classification is performed through a softmax layer:

5 $$\begin{aligned} \hat{y}_i = \arg \max _{c \in \mathcal {C}} P(y_i = c \mid f_{\theta }(\textbf{x}_i)) \end{aligned}$$

where $P(y_i = c \mid f_{\theta }(\textbf{x}_i))$ denotes the softmax probability that input $\textbf{x}_i$ belongs to class *c*, and $\mathcal {C}$ represents the complete set of gesture classes.

A key innovation in our method is the prototype learning branch, which complements the standard classification approach. For each class *c*, we define a prototype vector $\textbf{p}_c$ as the centroid of embeddings for its samples:

6 $$\begin{aligned} \textbf{p}_c = \frac{1}{|\mathcal {D}_c|} \sum _{\textbf{x}_i \in \mathcal {D}_c} f_{\theta }(\textbf{x}_i) \end{aligned}$$

where $\mathcal {D}_c$ represents the set of training samples for class *c*.

Unlike existing prototype learning, our method introduces architectural novelty by decoupling the prototype-based training process from the inference stage. During training, these prototypes serve as anchors that guide the organization of the feature space through a dedicated loss term. However, at inference time, we leverage the classification layer directly. This strategic decoupling enables efficient model operation while preserving the advantages of prototype-guided feature learning, particularly important in few-shot scenarios where prototype-based classification alone might be susceptible to noise and data sparsity.

The training process simultaneously optimizes two complementary terms: a cross-entropy loss $\mathcal {L}_{\text {CE}}$ that ensures discriminative power, and a prototype loss $\mathcal {L}_{\text {proto}}$ that enforces structural constraints on the embedding space.

The standard cross-entropy loss function is mathematically expressed as:

7 $$\begin{aligned} \mathcal {L}_{\text {CE}}(\textbf{x}_i, y_i)&= -\log P(y_i | \textbf{x}_i) \end{aligned}$$

8 $$\begin{aligned}&= -\log \text {softmax}(g_{\phi }(f_{\theta }(\textbf{x}_i)))_{y_i} \end{aligned}$$

where $g_{\phi }(\cdot )$ represents the classification layer with parameters $\phi$, and $P(y_i | \textbf{x}_i)$ indicates the model’s predicted probability that sample $\textbf{x}_i$ belongs to its ground truth class $y_i$. This formulation ensures robust learning of discriminative features while maintaining numerical stability during training.

The prototype loss component quantifies the similarity between each sample’s embedding and its corresponding class prototype using the Euclidean distance metric:

9 $$\begin{aligned} \mathcal {L}_{\text {proto}}(\textbf{x}_i, y_i) = d(f_{\theta }(\textbf{x}_i), \textbf{p}_{y_i}) \end{aligned}$$

where $d(\cdot , \cdot )$ denotes the Euclidean distance function, $f_{\theta }(\textbf{x}_i)$ represents the embedded feature vector of input sample $\textbf{x}_i$ generated by the 1D-CNN, and $\textbf{p}_{y_i}$ is the prototype vector corresponding to the true class label $y_i$. We selected the Euclidean distance metric after extensive experimentation, as it provides an optimal balance between computational efficiency and the ability to capture meaningful geometric relationships in the embedding space. This choice is supported by comprehensive comparative analyses against alternative distance metrics, as detailed in our ablation study.

The final hybrid loss function combines these components through a weighted sum:

10 $$\begin{aligned} \mathcal {L}_{\text {hybrid}} = (1 - \lambda ) \mathcal {L}_{\text {CE}} + \lambda \mathcal {L}_{\text {proto}} \end{aligned}$$

with a weighting parameter $\lambda$ controlling the trade-off between class discrimination and embedding space structure. This joint optimization enhances both accuracy and generalizability, enabling robust few-shot performance across diverse gesture contexts.

### Meta-learning for few-shot adaptation

A key challenge in EMG-based gesture recognition is the need to adapt models to new users with minimal training data. Traditional EMG gesture recognition systems often require extensive retraining when adapting to new conditions, limiting their practical applicability. To address this, we integrate meta-learning with our prototype learning framework to enable rapid adaptation using just a few examples.

We leverage the computationally efficient Reptile algorithm ^61^, implementing an *N*-way *K*-shot classification setting, where the goal is to classify *N* gesture classes using only *K* observed samples per class. It operates in two main phases: (1) meta-training, where the model is adapted to a new task using a small number of examples, and (2) meta-testing, where the model is evaluated on a new task with unseen data. This two-phase approach allows the model to learn task-specific parameters and generalize to new tasks with minimal retraining.

**Algorithm 1:**
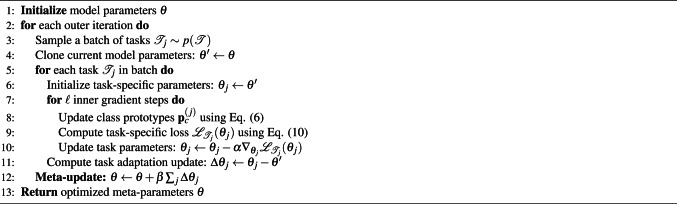
Meta-learning process

To process a meta-training dataset $\mathcal {D}_{\text {meta-train}}$ containing multiple *N*-way *K*-shot classification tasks $\mathcal {T}_j$, where we perform the following meta-training steps for each task: (1) sample *N* gesture classes with *K* examples per class, (2) initialize task-specific parameters $\theta _j$ from the global parameters $\theta$, (3) for each of the $\ell$ inner-loop steps, recalculate the class prototypes $\textbf{p}_c^{(j)}$ based on the task-specific parameters $\theta _j$ using Eq. (6) to ensure they reflect the updated feature space, (4) compute the prototype loss $\mathcal {L}_{\text {proto}}$ using these updated prototypes to reinforce intra-class similarity and inter-class separability, (5) calculate the task-specific loss $\mathcal {L}_{\mathcal {T}_j}(\theta _j)$ and update the task parameters through gradient descent: $\theta _j \leftarrow \theta _j - \alpha \nabla _{\theta _j} \mathcal {L}_{\mathcal {T}_j}(\theta _j)$, and (6) calculate the task-specific adaptation $\Delta \theta _j$ and use it to update global parameters during the meta-optimization step.

After processing multiple tasks, we update the model parameters $\theta$ to move it toward the task-adapted parameters through a first-order update:

11 $$\begin{aligned} \theta \leftarrow \theta + \beta \sum _j (\theta _j - \theta ) \end{aligned}$$

where $\beta$ is the meta-learning rate. We adopt a step size decay strategy to stabilize the training process, where the learning rate $\beta$ is progressively reduced during training:

12 $$\begin{aligned} \beta = \left( 1 - \frac{m}{M} \right) s \end{aligned}$$

where *m* and *M* denote the current and total meta-iterations, and *s* is the initial step size. This update encourages the model to learn an initialization that facilitates rapid adaptation across different tasks.

A key innovation of our approach is the tight coupling between prototype computation and task adaptation. After each inner-loop step, prototypes are dynamically recomputed to align with the evolving task-specific feature space. This ensures prototypes act as adaptive anchors that capture both stable inter-task patterns and task-specific variations. The full procedure is summarized in Algorithm 1.

## Experiments

In this section, we present a comprehensive evaluation of our proposed deep prototype learning framework for EMG-based gesture recognition. We first describe the experimental datasets, followed by detailed experimental settings. We then analyze our model’s performance through extensive comparisons with state-of-the-art methods across multiple evaluation protocols, including cross-session and cross-user generalization. Finally, we conduct an in-depth analysis of computational efficiency and ablation studies to validate our design choices.

**Table 1:** Characteristics of the EMG datasets used in our experiments. All datasets are from the Ninapro database collection, with varying numbers of subjects, gestures, and recording configurations.

Dataset Properties	DB1 ^55^	DB2 ^55^	DB3 ^55^	DB4 ^62^	DB5 ^62^
Number of Subjects	27	40	11 (Amputee)	10	10
Number of Gestures	53	50	50	53	53
Repetitions per Gesture	10	6	6	6	6
EMG Channels	10	12	12	12	16
Original Sampling Rate	100 Hz	2000 Hz	2000 Hz	2000 Hz	200 Hz
Downsampled Rate	–	100 Hz	100 Hz	100 Hz	–

### Datasets

Our experiments are conducted on Ninapro DB1-DB5 ^55,62^, which are widely recognized benchmarks for EMG-based gesture classification research. Each dataset includes 50-53 distinct gesture types performed by at least 10 subjects, with DB3 specifically focusing on amputee patients (detailed in Table 1). Following ^15^, we preprocess all datasets using a 200 ms window size with 10 ms increments, with DB2 to DB4 datasets downsampled to 100 Hz.

We train and evaluate our models using both cross-session and cross-user protocols, following established practices in gesture recognition and domain adaptation ^15,44,55^. Specifically, for cross-session evaluation, we follow the inter-session protocols of Atzori *et al.* ^11,55^. In DB1, sessions 2, 5, and 7 serve as test sets, while in DB2-DB5, sessions 2 and 5 are used as test sets. To enable few-shot adaptation, a small number of samples are drawn from the designated test sessions. These samples are used only for prototype adaptation and do not constitute conventional training data, allowing the model to adjust to session-specific variations such as electrode placement shifts or muscle fatigue without violating the cross-session evaluation protocol. By selectively using a few representative samples from the target session, we capture temporal variability while preserving the integrity of testing, demonstrating the model’s ability to generalize from limited target-domain data rather than relying on extensive retraining. For cross-user evaluation, we employ leave-one-subject-out cross-validation, where we iteratively test on one subject while training on the others ^15,44,63^. On the left-out subject, the test sessions are 2, 6, 7, 8, and 10 for DB1, and sessions 2, 5, and 6 for DB2 to DB5. The remaining sessions are used as the few-shot adaptation sets. The final performance is computed as the average across all cross-validation iterations.

### Experimental settings

For the few-shot adaptation, the training and testing sets are partitioned into meta-training ($\mathcal {D}_{\text {meta-train}}$) and meta-testing ($\mathcal {D}_{\text {meta-test}}$) sets, respectively. To construct each task $\mathcal {T}_j$, we randomly sample *N* classes from the corresponding meta-set ($\mathcal {D}_{\text {meta-train}}, \mathcal {D}_{\text {meta-test}}$), ensuring diversity across tasks. The training set consists of *K* randomly drawn instances per class across *N* gesture classes, while the test set contains 50 independently sampled instances per class to assess the model’s capacity to adapt to novel samples. We employ an inner-loop optimization to fine-tune task-specific parameters and an outer-loop update for meta-learning, then evaluate generalization on unseen test data using meta-testing. The meta-training procedure consists of $M = 200$ meta-iterations, with the initial step size set to $s=0.5$. During each training step, a single task is processed, consisting of $N \times K$ samples. The inner-loop optimization adapts the model to this task through 25 epochs of training, which in our setting corresponds to 25 gradient updates since the batch size covers the entire support set. This procedure consistently ensures convergence across tasks. All network weights are initialized using the Glorot (Xavier) uniform scheme ^64^.

The model is optimized using the Adam optimizer with a fixed learning rate of 0.001. The loss function defined in Eq. (10) employs a default weighting parameter $\lambda =0.25$, with the impact on the model performance of varying $\lambda$ analyzed in the Ablation Section. During meta-testing, the model is evaluated on previously unseen tasks sampled from $\mathcal {D}_{\text {meta-test}}$, and the test-set performance is computed after adaptation. All experiments were implemented in Python (v3.10.16) using TensorFlow (v2.10.0). GPU acceleration used CUDA (v11.2) and cuDNN (v8.7). Experiments were conducted under two hardware environments: (1) Intel i7-12700H CPU with 32 GB RAM and an NVIDIA RTX 3080Ti GPU with 16 GB VRAM, and (2) Intel i9-12900KS CPU with 32 GB RAM and an NVIDIA RTX 3070Ti GPU with 8 GB VRAM.

**Table 2:** Cross-session gesture classification accuracy (unit: %), with the highest performance highlighted in bold. The values in parentheses next to the performance indicate the number of classes used. The final four rows present a comparison with the few-shot learning method for a fair evaluation, following experiment 1 in^45^ with the 5-way classification setting, where the 5 classes are randomly sampled from the full set of gestures for each task.

Method	Input	Model	DB1	DB2	DB3	DB4	DB5
Zabihi*et al.* ^65^	Raw signal	Transformer	–	86.2 (49)	–	–	–
Zhang *et al.* ^28^	Features	1D-CNN + LSTM	89.7 (53)	91.7 (50)	–	–	–
Murugiah *et al.* ^24^	Raw signals	Ensemble	92.9 (53)	90.8 (50)	–	–	–
AtzoriNet ^11^	Raw signals	2D-CNN	66.6 (53)	60.2 (50)	39.1 (50)	–	–
Atzori *et al.* ^55^	Features	Random Forest	75.3 (53)	74.2 (50)	47.8 (50)	–	–
DVMSCNN ^23^	Raw signals	2D-CNN	86.7 (53)	83.3 (50)	70.6 (50)	73.3 (53)	–
EMGHandNet ^19^	Features	1D-CNN + BiLSTM	95.8 (52)	**95.9** (49)	–	91.6 (52)	–
MSCNet ^21^	Raw signals	2D-CNN	83.2 (53)	82.9 (50)	69.2 (50)	71.7 (53)	–
MyoCNN ^22^	Raw signals	2D-CNN	78.3 (53)	79.2 (50)	67.2 (50)	68.1 (53)	–
MKCNN ^17^	Raw signals	2D-CNN	–	86.7 (50)	87.6 (50)	82.3 (53)	–
Zhang *et al.* ^66^	Raw signal	Transformer	–	81.5 (17)	–	–	88.2 (23)
Nguyen *et al.* ^67^	Raw signal	SDCT + Transformer	–	–	–	78.7(53)	89.2 (53)
MVCNN (+IMU) ^15^	Features	2D-CNN	–	94.4 (50)	87.1 (50)	–	91.3 (53)
Lee *et al.* ^20^	Features	2D-CNN	96.3 (53)	89.0 (50)	76.8 (50)	–	90.8 (53)
Pizzolato *et al.* ^62^	Features	Random Forest	65.6 (53)	73.2 (50)	–	69.1 (53)	69.0 (53)
GengNet ^12^	Raw signals	2D-CNN	78.9 (53)	59.4 (50)	57.0 (50)	67.4 (53)	78.9 (53)
DuNet ^13^	Raw signals	2D-CNN	79.4 (53)	52.6 (50)	41.3 (50)	64.8 (53)	77.9 (53)
HuNet ^14^	Raw signals	2D-CNN + LSTM	87.0 (53)	82.2 (50)	46.7 (50)	68.6 (53)	81.8 (53)
MVCNN ^15^	Features	2D-CNN	88.2 (53)	83.7 (50)	64.3 (50)	51.6 (53)	90.0 (53)
CMAM ^27^	Features	2D-CNN + LSTM + GAN	90.1 (53)	84.8 (50)	65.7 (50)	76.1 (53)	92.5 (53)
XceptionTime ^31^	Raw signals	1D-CNN	85.0 (53)	83.4 (50)	55.0 (50)	71.7 (53)	89.0 (53)
XCM ^32^	Raw signals	1D-CNN	90.5 (53)	84.8 (50)	65.0 (50)	78.1 (53)	94.0 (53)
sEMGXCM ^18^	Raw signals	2D-CNN	91.4 (53)	86.3 (50)	66.5 (50)	78.7 (53)	94.2 (53)
Ours (w/o adaptation)	Features	1D-CNN	**97.5** (53)	94.7 (50)	**91.3** (50)	**94.6** (53)	**97.6** (53)
FS-HGR (5-shot) ^45^	Raw signals	*Few-shot*, Dilated CNN	–	85.9 (5/50)	–	–	–
FS-HGR (10-shot) ^45^	Raw signals	*Few-shot*, Dilated CNN	–	89.7 (5/50)	–	–	–
Ours (5-shot)	Features	*Few-shot*, 1D-CNN	94.2 (5/53)	89.4 (5/50)	78.2 (5/50)	85.6 (5/53)	94.8 (5/53)
Ours (10-shot)	Features	*Few-shot*, 1D-CNN	97.7 (5/53)	94.9 (5/50)	86.9 (5/50)	91.2 (5/53)	97.7 (5/53)
Ours (20-shot)	Features	*Few-shot*, 1D-CNN	**98.4** (5/53)	**95.8** (5/50)	**91.3** (5/50)	**92.4** (5/53)	**98.1** (5/53)

**Table 3:** Cross-user gesture classification accuracy (%). Values in parentheses next to the performance denote the number of classes used, where the few-shot settings in the final row indicate a 5-way classification setting, where the 5 classes are randomly sampled from the full set of gestures for each task.

Method	Input	Model	DB1	DB2	DB3	DB4	DB5
Du *et al.* ^44^	Raw signals	Ensemble 2D-CNN	67.4 (52)	–	–	–	–
Ketyko *et al.* ^25^	Raw signals	LSTM	65.3 (12)	–	–	–	–
Tyacke *et al.* ^68^	Raw signals	Dilated efficient capsule network	–	78.3 (17)	–	–	–
Sun *et al.* ^69^	Raw signals	MSFEnet	–	86.2 (17)	–	–	–
Xie *et al.* ^26^	Raw signals	1D-CNN + RNN	–	63.7 (18)	–	–	–
Zhai *et al.* ^70^	Raw signals	2D-CNN	–	78.7 (49)			
Rahimian *et al.* ^71^	Raw signals	Dilated Causal CNN	–	92.5 (17)	–	–	–
Ding *et al.* ^72^	Raw signals	2D-CNN	–	78.7 (49)	–	–	–
Jiang *et al.* ^73^	Raw signals	2D-CNN + LSTM	–	87.9 (49)	–	–	–
NIMFT ^74^	Raw signals	1D-CNN + Transformer	–	91.9 (49)	–	–	–
Fatayer *et al.* ^75^	Features	2D-CNN	–	87.9 (41)	60.7 (41)	–	–
CNN (spectrogram) ^16^	Features	2D-CNN	–	73.1 (49)	66.3 (49)		
Lehmler *et al.* ^76^	Raw signals	1D-CNN	–	67.8 (17)	52.3 (17)	54.6 (12)	–
MKCNN ^17^	Raw signals	2D-CNN	–	63.6 (50)	76.8 (50)	60.1 (53)	–
TL-MKCNN ^17^	Raw signals	2D-CNN	–	65.3 (50)	82.5 (50)	62.3 (53)	–
Wang *et al.* ^41^	Raw signals	2D-CNN	40.2 (50)	–	–	–	52.7 (53)
Wang *et al.* ^63^	Features	SVM, Random Forest	–	–	–	84.0 (10)	93.5 (10)
MVCNN ^15^	Features	2D-CNN	86.0 (53)	81.5 (50)	57.5 (50)	56.5 (53)	88.0 (53)
Ours (Proto-TL)	Features	1D-CNN	95.5 (53)	93.0 (50)	86.4 (50)	84.3 (53)	94.8 (53)
Ours (20-Shot)	Features	*Few-shot*, 1D-CNN	63.8 (5/53)	63.5 (5/50)	49.6 (5/50)	65.7 (5/53)	69.4 (5/53)

### Results

In this section, we present the experimental results for cross-user and cross-session gesture classification accuracies, comparing our proposed approach with state-of-the-art methods.

#### Cross-session generalization

Table 2 presents a cross-session evaluation of our model under two settings: standard classification without adaptation and few-shot learning with adaptation. This dual setting allows us to assess both the base generalization of our deep prototype learning framework and its adaptability in data-scarce scenarios.

In the standard setting, our model (Ours w/o adaptation) achieves the highest accuracy across DB1 (97.5%), DB3 (91.3%), DB4 (94.6%), and DB5 (97.6%), outperforming existing methods by 1–3%. Although EMGHandNet ^19^ slightly outperforms ours on DB2 (+1.3%), it omits the resting state class, unlike most of the compared models. Moreover, our model achieves competitive performance without relying on BiLSTM or 2D CNNs, benefiting instead from efficient feature selection and a prototype-based architecture. These design choices not only reduce computational cost but also yield robust accuracy across various datasets, highlighting the model’s practical applicability.

Further, the few-shot learning evaluation provides strong evidence of our model’s generalization capabilities. Compared to the state-of-the-art FS-HGR ^45^ under identical experimental conditions, our approach achieves +5.2% accuracy in the 10-shot setting and +3.5% in the 5-shot setting on DB2. These improvements are notable given the inherent difficulty of few-shot learning, where models must adapt to new patterns with minimal data. We attribute this performance advantage to three key aspects of our framework: (i) averaged cepstrum coefficients, which capture EMG signal characteristics robustly; (ii) prototype-based learning, which provides effective and discriminative feature representations; and (iii) an efficient meta-learning strategy, enabling rapid adaptation to unseen sessions. Together, these components facilitate consistent performance gains across different shot settings, a conclusion further supported by inter-session experiments with simpler classifiers on the CCA feature set (reported in Supplementary Information, Supplementary Fig. S2), which demonstrate the effectiveness of our feature design in improving cross-session generalization.

#### Cross-user generalization

Table 3 compares our framework with state-of-the-art cross-user adaptation ^15,25,44,63^ and transfer learning methods ^17,68,76,77^. To enable fair comparison, we additionally implement a transfer learning variant of our prototype-based model (Proto-TL). In this setting, the source domain corresponds to the labeled data of training subjects, while the target domain corresponds to data from previously unseen test subjects, consistent with the cross-user protocol defined and widely used in the Experimental Settings Section. Proto-TL employs the same network architecture and hyperparameters as our few-shot setup: it is first trained on source data for 20 epochs, and subsequently adapted to the target domain for 60 epochs, following established protocols ^15,44^.

Proto-TL consistently outperforms existing methods, achieving 1-9% accuracy gains on DB1–DB3 and DB5, and a notable 21% improvement on DB4 compared to MVCNN ^15^. These improvements are likely influenced by the combination of prototypical learning and our CCA feature set, which provides informative EMG representations. As shown in the Supplementary Fig. S2, experiments with simpler classifiers (Linear Discriminant Analysis, Logistic regression, and Naive Bayes) indicate that CCA features inherently excel at capturing representations that enhance generalization across subjects, sessions, and datasets. Unlike many prior approaches relying on complex multi-feature fusion ^15–17,75^, our model achieves strong performance using only these univariate CCA features, highlighting the effectiveness of carefully engineered inputs over high-dimensional representations.

Furthermore, in the few-shot (20-shot) setting, our method demonstrates strong adaptability with significantly less data than traditional approaches. It achieves competitive accuracy across datasets, particularly excelling on DB4 and maintaining robust performance on DB3 despite the challenges of amputee data. These results underscore the efficiency and practicality of our approach for user-independent EMG gesture recognition with minimal calibration data.

**Table 4:** Comparison of computational efficiency between our 1D-CNN model and conventional 2D-CNN models across five datasets in the cross-user setting. Metrics include averaged training time (in seconds) and peak memory usage (in megabytes). The results demonstrate that our 1D-CNN achieves consistently lower training time and significantly reduced memory consumption.

	DB1	DB2	DB3	DB4	DB5
*Averaged training time (Sec)*					
1D-CNN (Ours)	809.3	761.0	760.5	778.0	848.3
2D-CNN	871.8	804.1	812.3	832.2	876.0
*Maximum memory cost (MB)*					
1D-CNN (Ours)	37.0	38.1	38.1	38.1	39.7
2D-CNN	149.6	140.5	132.1	135.6	147.4

**Table 5:** Comparison of computational efficiency between prototype-based Reptile and MAML meta-learning approaches across five datasets in the cross-session setting (5-way, 5-shot). Metrics include averaged training time (in seconds) and peak memory usage (in megabytes). The results highlight the superior computational and memory efficiency of our Reptile-based method.

	DB1	DB2	DB3	DB4	DB5
*Averaged training time (Sec)*					
Reptile (Ours)	759.6	787.9	776.1	762.9	833.1
MAML	995.2	1029.1	1018.0	1030.1	1085.0
*Maximum memory cost (MB)*					
Reptile (Ours)	37.0	38.1	38.1	38.1	39.7
MAML	67.4	69.3	71.0	71.6	84.0

#### Efficiency analysis

A key advantage of our framework is its computational efficiency, essential for real-time gesture recognition in resource-limited settings. This efficiency stems from the lightweight 1D-CNN architecture combined with optimized feature design, reducing both computational cost and memory usage compared to common 2D-CNN methods. To ensure a fair comparison, the 2D-CNN baseline was implemented using the same architecture as our 1D-CNN (described in Supplementary Table S1), with the kernel dimension expanded from 1D (size 3) to 2D (size 3$\times$3), while keeping the layer sizes and strides identical. The cross-subject protocol was chosen for this comparison because it exposes differences in representational efficiency under strong inter-subject variability. Table 4 compares training efficiency between our 1D-CNN and conventional 2D-CNN models trained under a cross-user protocol. Our 1D-CNN reduces training time by 3-7% and cuts memory usage by approximately 75%, representing a 4$\times$ improvement.

Table 5 compares our Reptile-based few-shot prototype learning (EMG-Adapt) to a MAML-based method in a cross-session setting. This protocol directly measures a meta-learner’s ability to adapt to temporal non-stationarity within the same individual, such as signal amplitude drift or electrode displacement, rather than structural variability across users. EMG-Adapt achieves 23-25% faster training and uses 45-52% less memory across datasets, benefiting from first-order gradient updates that avoid the higher overhead of MAML’s second-order derivatives ^61^. These efficiency gains are especially pronounced on DB5, where additional channels increase computational demands.

Moreover, we compare inference complexity using Big-O notation. For a *D*-layer 1D-CNN the forward pass requires $O(\sum _{\forall l}L_{l}k_{l}C_{in,l}C_{out,l})$, which with our per-sample length $L=1$ simplifies to $O(\sum _{\forall l}k_{l}C_{in,l}C_{out,l})$ where *k* denotes kernel size, *C* is number of input channels, and *l* is layer index. By contrast, a 2D-CNN has complexity $O(\sum _{\forall l}H_{l}W_{l}k_{h,l}k_{w,l}C_{in,l}C_{out,l})$; when a 2D baseline reshapes input vectors into a spatial map (e.g., height $H=C$, width $W=$ feature set) the per-layer cost ratio between 2D and 1D is approximately $\tfrac{k_h k_w}{k}$ with a 2D kernel is approximately squared times heavier than its 1D counterpart. A similar contrast emerges in meta-learning. During inference, both MAML and Reptile require the same forward-pass cost as the underlying CNN. However, if test-time adaptation is employed, MAML necessitates *S* inner-update steps, each involving forward and backward passes, leading to an additional *O*(*S*) multiplicative factor. In practice, this overhead can dominate inference time for moderate *S*. Reptile, as used in our setting, does not require such test-time updates and therefore maintains the lightweight complexity of a single forward pass. Collectively, these observations highlight two efficiency advantages of our approach: (i) using a 1D-CNN reduces per-layer complexity by up to a factor of three relative to comparable 2D-CNN baselines (as well as unnecessary manual feature extraction), and (ii) adopting Reptile eliminates the adaptation overhead incurred by MAML at deployment.

Overall, these results demonstrate that our approach delivers strong performance while substantially reducing resource requirements, making it well-suited for scalable, real-time, and embedded EMG gesture recognition applications.

### Ablation studies

To understand the contributions of individual components within our framework and guide future improvements in EMG-based gesture recognition, we conduct targeted ablation studies. These investigations isolate and analyze the effect of specific design choices, including input feature selection, the number of training samples per class, the balance between prototype and classification losses, and the structure of learned prototypes. By systematically varying each component while holding others constant, we aim to clarify their roles in enabling generalization, efficient learning, and robust cross-session performance.

#### Feature selection

To comprehensively evaluate the discriminative power of different EMG signal features, we conducted a systematic comparison between our proposed CCA and 26 other time- and frequency-domain features commonly used in EMG analysis. Drawing from established literature ^15,22,53,54,78^, we examined features across multiple categories:

- Time-domain features: mean absolute value (MAV), waveform length (WL), Willison amplitude (WAMP), MAV slope (MAVS), root mean square (RMS), slope sign changes (SSC);
- Statistical features: mean square (MSQ), v-order 3 (V3), log detector (LD), difference absolute standard deviation (DABS);
- Complexity measures: maximum fractal length (MFL), myopulse percentage rate (MPR);
- Frequency-domain features: mean frequency (MNF), power spectrum ratio (PSR);
- Model-based features: four autoregressive coefficients (ARC);
- Cepstrum features: three cepstrum coefficients (CC), average of cepstrum coefficients (CCA);
- Time-frequency features: two discrete wavelet transform coefficients (DWTC), three discrete wavelet transform packet coefficients (DWTPC).

To ensure an unbiased evaluation of each feature’s inherent discriminative capability, we employed our baseline 1D-CNN architecture without prototype learning. The model processed each feature independently with an input dimension of $e \times 1$, where *e* denotes the number of electrode channels. All experiments maintained consistent training parameters: 20 epochs, 0.002 learning rate, and the Adam optimizer.

The comparative analysis presented in Table 6 reveals several key insights. Most notably, CCA emerged as the superior feature across all five datasets, consistently achieving the highest classification accuracy. This exceptional performance can be attributed to three key factors: First, CCA effectively captures subtle variations in the EMG signal’s spectral composition, providing rich discriminative information for gesture classification. Second, by averaging cepstral coefficients across channels, CCA creates a more stable representation that is less sensitive to electrode-specific variations. Third, CCA demonstrates remarkable resilience to dataset shifts and session-specific variations, maintaining consistent performance across different recording sessions and experimental conditions. We further validate the effectiveness of the proposed CCA feature by comparing gesture recognition performance using three simpler classifiers (LDA, logistic regression, and Naive Bayes) applied to univariate features (Supplementary Fig. S2). While Table 6 and Supplementary Fig. S2 report single-feature performance, and many prior studies typically employ feature ensembles ^55,59,79,80^, these results demonstrate that CCA alone provides strong and consistent discriminative power. Collectively, these findings highlight CCA as a highly informative and robust feature, making it particularly well-suited for EMG-based gesture recognition, where signal quality and user-specific characteristics may vary substantially.

**Table 6:** Gesture classification accuracy in the cross-session setting using univariate feature inputs (unit: %). Features are ranked by average performance across all databases, with the highest-performing feature for each dataset highlighted in bold.

No.	Feature	DB1	DB2	DB3	DB4	DB5	Average
1	CCA	**96.3**	**91.6**	**86.8**	**92.2**	**94.5**	**92.3**
2	WL	90.4	65.8	62.1	90.9	86.7	79.1
3	DWTPC1	95.5	63.5	53.5	85.7	94.3	78.5
4	MAV	95.4	60.7	42.4	92.7	94.3	77.1
5	CC1	87.1	77.9	69.3	75.3	73.4	76.6
6	DWTC1	95.4	62.9	39.4	90.4	94.4	76.5
7	MNF	71.5	58.6	58.0	58.7	38.7	57.1
8	LD	95.0	34.8	23.4	22.7	94.2	54.0
9	V3	52.6	2.1	2.2	90.6	88.1	47.1
10	MSQ	84.6	2.4	2.3	51.2	93.2	46.7
11	SSC	95.2	21.9	12.2	3.4	94.2	45.4
12	DABS	68.3	4.9	3.6	85.6	42.6	41.0
13	PSR	74.3	23.3	24.9	24.3	40.4	37.4
14	MFL	78.5	23.4	16.8	21.7	40.5	36.2
15	MAVS	88.3	3.7	2.7	23.5	58.7	35.4
16	DWTPC2	85.2	6.5	3.2	21.6	58.4	35.0
17	DWTPC3	76.2	6.1	3.2	21.2	59.3	33.2
18	MPR	2.2	17.0	13.4	92.2	2.4	25.4
19	ARC1	72.1	5.6	5.5	6.0	35.2	24.9
20	CC3	77.5	5.0	5.0	5.1	30.5	24.6
21	CC2	76.1	4.6	4.7	4.9	31.4	24.3
22	WAMP	37.3	2.9	2.9	3.5	60.4	21.4
23	ARC4	66.2	4.7	4.5	5.0	25.2	21.1
24	RMS	10.0	2.4	2.1	62.7	25.9	20.6
25	DWTC2	7.3	3.2	2.6	22.7	20.9	11.3
26	ARC2	24.2	5.6	5.5	6.2	5.0	9.3
27	ARC3	24.9	4.2	4.4	4.9	4.9	8.7

#### Effect of *K* on Few-shot adaptation

To analyze the performance of few-shot adaptation, we evaluated varying numbers of training samples per class (*K*) in a 5-way cross-session classification task using five Ninapro datasets. Specifically, we evaluate five learning shot settings (1-shot, 2-shot, 5-shot, 10-shot, and 20-shot) within a 5-way classification framework under a cross-session setup where the final session is designated as the test set, while the remaining sessions serve as training data. In the 1-shot setting, we set $\lambda = 1$ because prototype-based learning is inherently limited when only a single sample per class is available, making effective representation learning and comparison challenging. In contrast, for all other settings, we set $\lambda = 0.25$ to effectively balance the contribution of the prototype-based loss, ensuring that the model leverages both class prototypes and discriminative features for enhanced generalization.

As shown in Supplementary Table S2, classification accuracy improved consistently with increasing *K*, indicating a strong positive correlation between sample size and model performance. Notably, performance gains were most pronounced between 1-shot and 2-shot settings, with accuracy increases up to 46%, underscoring the critical role of early-shot information in prototype learning. The model achieved its highest accuracy at 20-shot, ranging from 92.1% (DB3) to 97.8% (DB5), suggesting that 20 samples per class are sufficient for robust representation. While DB3 (amputee data) showed the lowest absolute accuracy, it exhibited a substantial relative improvement (56.7%), highlighting the model’s adaptability to complex EMG signals with more data. These findings confirm the effectiveness of our prototype-based framework under limited supervision and across diverse datasets.

#### Impact of loss balancing

The effectiveness of our framework heavily depends on finding an optimal balance between the prototype loss and the cross-entropy loss to maximize both generalization capability and class discrimination. To systematically analyze this relationship, we investigated the effect of balancing prototype loss and cross-entropy loss through the weighting parameter $\lambda$ in a 5-shot 5-way classification setting. As shown in Supplementary Table S3, performance peaked consistently at $\lambda = 0.25$ across most datasets, indicating that combining prototype learning with moderate discriminative supervision yields the most effective representation. Compared to using prototype loss alone ($\lambda = 0$), this configuration improved accuracy by over 25–51%, while larger values of $\lambda$ led to gradual performance degradation. Notably, relying solely on prototype loss resulted in a significant accuracy drop ($\sim$40%), suggesting that prototype learning alone cannot provide adequate class separation. Conversely, excessive emphasis on cross-entropy loss weakened the model’s ability to structure feature space meaningfully. These findings underscore the importance of synergy between the two loss terms, where prototype loss encourages generalizable structure and cross-entropy enforces discriminability. The consistency of results across five EMG datasets supports the robustness of our loss balancing strategy and its relevance for real-world gesture recognition applications.

#### Prototype analysis

**Fig. 2:**
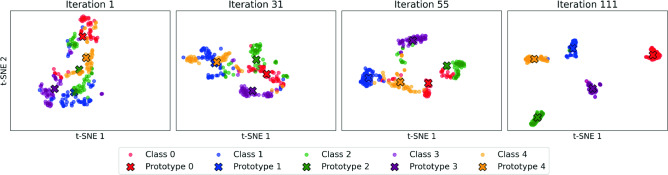
Visualization of prototype convergence during meta-training. Sub-figures (a–d) show the evolution of class prototypes in the embedding space across training iterations. The visualizations were produced using t-SNE applied to the learned embeddings, all on the same scale. Initially, the prototypes appear dispersed and overlapping; over successive iterations they converge into compact, well-separated clusters, indicating improved feature space organization. This refinement correlates with cross-session classification accuracy improvements: 25% at meta-iteration 1 (**a**), 50% at meta-iteration 31 (**b**), 75% at meta-iteration 55 (**c**), and 97% at meta-iteration 111 (**d**) on the Ninapro DB5, Subject 1 dataset.

To validate the effectiveness of our prototype-based learning approach, we conduct an in-depth analysis of prototype evolution and feature space structuring throughout the meta-learning process under a 20-shot cross-session setting. As shown in Fig. 2, visualization on Ninapro DB5 reveals that as training progresses, class prototypes gradually form distinct, well-separated clusters with improved intra-class compactness, mirroring increases in test accuracy from 25% to 97%. This progression highlights the model’s ability to structure the feature space meaningfully through prototype refinement.

To complement this qualitative finding, we further examined how the choice of similarity metric in the prototype loss (Eq. 9) affects model performance. Four representative formulations: Cosine similarity, Euclidean distance, Mahalanobis distance, and Kullback–Leibler (KL) divergence were compared across all five Ninapro databases (Supplementary Table S4). Euclidean distance consistently achieves superior performance, with notable margins of improvement: +1.3% over Cosine similarity on DB1, +1.6% on DB2, +1.5% on DB3, +3.2% on DB4, and +1.8% on DB5. The substantial performance gap between Euclidean distance and Mahalanobis distance (over 30% across all datasets) is particularly noteworthy, suggesting that simpler distance metrics may be more robust for EMG-based gesture classification.

The superior performance of Euclidean distance can be attributed to several factors. First, its ability to preserve the natural topology of the feature space allows for more intuitive prototype positioning. Second, unlike Mahalanobis distance, it does not assume specific data distribution characteristics, making it more robust to the non-Gaussian nature of EMG signals. Third, compared to KL divergence, its symmetric property ensures consistent prototype-to-sample relationships, which are crucial for stable learning. These characteristics make Euclidean distance particularly effective for capturing the geometric relationships between prototypes in high-dimensional space while maintaining computational efficiency.

### Real-world feasibility

To evaluate the practicality of the proposed EMG-Adapt framework for real-time deployment, we conducted an extensive inference benchmarking analysis. Inference performance was measured over 500 runs (including 50 warm-up runs) using a batch size set to 1, capturing mean, median, and standard deviation of latency, model size, peak memory usage, FLOPs, and throughput. As summarized in Supplementary Table S6, the model exhibits stable and low inference latency (7–15 ms) across both a development workstation (Intel Core i9-12900KS CPU with 32 GB RAM and an NVIDIA RTX 3070Ti GPU with 8 GB VRAM) and an ARM-based device (Apple M2 CPU, 8-core, 8 GB RAM, macOS 15.6, Python v3.13.0). The throughput exceeded 130 samples/s on the ARM platform, indicating that the model can sustain real-time EMG decoding even under computationally constrained environments. Model footprint remained compact (<3 MB) with low memory demand (<1 MB peak), further supporting portability and embedded deployment. These results confirm real-time inference feasibility, validating that the model executes on-device without latency bottlenecks. However, we distinguish this from continuous online adaptation, a distinct objective reserved for future work.

Beyond computational efficiency, the overall design of EMG-Adapt facilitates clinical and assistive integration. Its low-latency inference and compact architecture enable seamless interaction with wearable EMG interfaces, prosthetic controllers, and rehabilitation devices without the need for server-based processing. Moreover, the few-shot adaptation mechanism reduces the dependency on extensive calibration, allowing users to personalize gesture decoding using a small number of samples. These characteristics jointly demonstrate that EMG-Adapt is not only algorithmically effective but also operationally viable for real-world and clinical deployment scenarios.

## Conclusion

In this paper, we introduced EMG-Adapt, a novel few-shot prototype adaptation framework designed to improve the robustness and data efficiency of EMG-based gesture recognition models by combining the representational power of prototype learning with the rapid adaptation capabilities of meta-learning. The framework’s key technical contributions include: (i) a CCA feature extraction method that is less sensitive to noise and inter-session variability, (ii) a deep prototype learning model with a hybrid loss function that optimizes both discriminative classification and a well-structured embedding space, and (iii) a meta-learning-based prototype update strategy enabling efficient domain adaptation with only a few labeled examples.

Through extensive experiments on five public EMG datasets, we demonstrated that EMG-Adapt achieves state-of-the-art performance in both cross-session and cross-user generalization scenarios, while maintaining computational efficiency. Notably, the CCA feature drives these improvements by providing robust, noise-resistant representations that facilitate stable adaptation even under high signal variability. These results highlight the framework’s advantages, including reduced calibration requirements, improved generalization across users and sessions, and lower computational cost compared to conventional 2D-CNN-based approaches.

At the same time, we note the following areas for future studies: First, the framework has been evaluated primarily on offline datasets; an interesting extension is real-time deployment which may be subject to additional challenges such as latency and sensor noise. Second, while cross-user evaluation demonstrates generalizability, further testing on entirely unseen datasets or in more diverse real-world scenarios can further assess robustness. Finally, the current model focuses on a limited set of gestures; although this aligns with common practice in studies using Ninapro databases (typically 10–53 gestures depending on the exercise set), it should explore scalability to a much larger gesture vocabulary (e.g., 100 or more gestures).

Future work will explore how the distribution of few-shot adaptation samples across sessions influences performance, as analyzing adaptation over sequential sessions may provide valuable insights for real-world deployment. In addition, we plan to investigate self-supervised and continuous learning strategies to further reduce the dependency on labeled data and enable online adaptation. Expanding the framework to support a larger number of gestures and real-time deployment will also be pursued to enhance its practical applicability in prosthetics, assistive devices, and human-computer interaction systems.

## Supplementary Information

Supplementary Information.

## Data Availability

This study uses the publicly available Ninapro datasets (DB1-5), which can be accessed at: https://ninapro.hevs.ch
