# Supplementary material for: Few-shot prototype adaptation for generalizable electromyography gesture recognition
Source: Sci Rep. 2026 Mar 7;16:12546. doi: 10.1038/s41598-026-40352-6 (PMC13086972; doi:10.1038/s41598-026-40352-6)
Supplement: Supplementary file 1 — Supplementary Information. [file 41598_2026_40352_MOESM1_ESM.pdf]

Supplementary Information

Cepstral Coefficients

Definition and Mathematical Formulation

Cepstral coefficients[1–4] provide a compact representation of the spectral characteristics of a signal by taking the inverse Fourier transform (IFT) of the logarithm of its magnitude spectrum. For a discrete-time sEMG signal $x[n]$ of length N, the computation is as follows:

1. Discrete Fourier Transform (DFT):

$$X[k] = \sum_{n=0}^{N-1} x[n] e^{-j2\pi kn/N}, \quad k = 0, 1, \ldots, N-1 \tag{1}$$

2. Logarithm of the magnitude spectrum:

$$L[k] = \log\left|X[k]\right|, \quad k = 0, 1, \ldots, N-1 \tag{2}$$

3. Inverse DFT to obtain the cepstral coefficients:

$$c[n] = \frac{1}{N} \sum_{k=0}^{N-1} L[k]\, e^{j2\pi kn/N}, \quad n = 0, 1, \ldots, N-1 \tag{3}$$

Where:

- $x[n]$ is the sEMG signal in the time domain.
- $X[k]$ is the DFT of $x[n]$.
- $L[k]$ is the logarithm of the magnitude spectrum.
- $c[n]$ is the cepstral coefficient sequence.
- Note that FFT length is identical to the signal length.

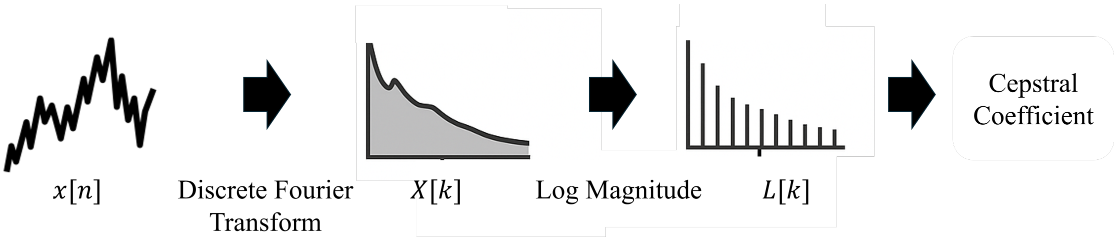

Figure S1. Cepstral coefficients

Quefrency and Interpretation

The term **quefrency**[1;2] refers to the "time-like" domain obtained after taking the inverse Fourier transform of the log spectrum. Each index n in $c[n]$ corresponds to a quefrency value, which indicates the periodicity of spectral components in the original signal (Supplementary references 1-4).

Key points are as follows:

- Low quefrency coefficients capture the overall spectral envelope, representing slow variations in muscle activation patterns.
- High quefrency coefficients capture fine spectral details or rapid fluctuations, which are often related to noise or transient artifacts in sEMG signals.
- By averaging cepstral coefficients across sliding windows, the low-to-mid quefrency features dominate, providing a stable and generalizable representation.

Signal Processing Perspective and Advantages

From a signal processing perspective, cepstral coefficients efficiently encode the spectral envelope of sEMG signals, which captures the underlying muscle activation patterns while suppressing high-frequency variations due to noise or transient artifacts. Specifically:

1. Noise suppression: By working in the log-spectral domain, cepstral coefficients diminish the impact of instantaneous noise spikes that commonly occur in sEMG measurements.
2. Dimensionality reduction: Averaging cepstral coefficients over time windows produces a low-dimensional representation that preserves key spectral features, reducing computational burden for classifiers without losing discriminative power.
3. Robust temporal-spectral representation: Despite each sliding window only partially capturing a gesture, the averaged cepstral coefficients accumulate the essential spectral characteristics across multiple windows, providing a stable feature representation.

Generalization Across Subjects and Sessions

SEMG signals are highly variable across individuals and recording sessions due to factors such as electrode placement, skin impedance, and muscle fatigue. Averaged cepstral coefficients are particularly effective for generalization because:

- They summarize the global spectral structure rather than relying on transient amplitude details.
- They are less sensitive to timing misalignments or minor differences in gesture execution speed.
- By averaging across sliding windows, they mitigate session-specific noise while retaining discriminative information relevant to gesture identity.

Supplementary Tables

Layer	Filters / Kernel Size / Stride	Output Shape
Input	-	(None, e, 1)
BatchNorm	-	(None, e, 1)
Conv1D	64 / 3 / 1	(None, $e-2$, 64)
BatchNorm	-	(None, $e-2$, 64)
Conv1D	64 / 3 / 1	(None, $e-4$, 64)
BatchNorm	-	(None, $e-4$, 64)
Conv1D	64 / 1 / 1	(None, $e-4$, 64)
BatchNorm	-	(None, $e-4$, 64)
Conv1D	64 / 1 / 1	(None, $e-4$, 64)
BatchNorm	-	(None, $e-4$, 64)
Flatten	-	(None, $(e-4)\times 64$)
Dense	512	(None, 512)
BatchNorm	-	(None, 512)
Dense	512	(None, 512)
BatchNorm	-	(None, 512)
Dense	128	(None, 128)
BatchNorm	-	(None, 128)
Dense	classes	(None, classes)

Table S1. 1D-Convolutional Neural Network Architecture: Baseline model configuration employing 1D convolutions, where e denotes the number of input electrode channels.

	DB1	DB2	DB3	DB4	DB5
**Inter-session**					
Ours (w/o adaptation)	97.5 (±1.8)	94.7 (±3.2)	91.3 (±6.1)	94.6 (±2.7)	97.6 (±1.5)
Ours (5-shot)	88.2 (±3.1)	89.4 (±3.0)	85.5 (±6.3)	88.6 (±3.8)	91.8 (±2.1)
Ours (10-shot)	94.6 (±2.3)	93.3 (±2.5)	89.6 (±7.1)	92.2 (±4.5)	95.1 (±1.9)
**Inter-subject**					
Ours (Proto-TL)	95.5 (±2.8)	93.0 (±4.7)	86.4 (±4.5)	84.3 (±7.5)	94.8 (±3.0)
Ours (20-shot)	63.8 (±10.7)	63.5 (±4.2)	49.6 (±4.9)	65.7 (±9.0)	69.4 (±7.9)

Table S2. Mean ± standard deviation of model performance (%) on Ninapro DB1–DB5 datasets over five runs with different random seeds, for both inter-session and inter-subject evaluation settings.

K	DB1	DB2	DB3	DB4	DB5
1	40.0 (±5.6)	36.3 (±4.5)	35.4 (±6.2)	66.0 (±5.1)	42.8 (±3.5)
2	77.1 (±6.1)	82.1 (±7.5)	64.3 (±9.1)	67.2 (±6.5)	79.4 (±7.3)
5	93.2 (±4.8)	89.4 (±3.0)	80.5 (±8.4)	86.6 (±9.5)	95.3 (±3.1)
10	97.6 (±2.3)	95.2 (±2.1)	86.8 (±6.6)	91.9 (±7.2)	97.1 (±1.2)
20	**98.1** (±1.2)	**95.5** (±1.4)	**92.1** (±3.2)	**92.7** (±6.5)	**97.8** (±0.8)

Table S3. Classification accuracy comparison for few-shot learning across different numbers of training samples per class (1-shot to 20-shot) in a 5-way classification setting. Results show cross-session adaptation performance on Ninapro DB1-DB5 datasets (unit: %).

λ	DB1	DB2	DB3	DB4	DB5
0	41.7 (±4.3)	43.8 (±7.1)	35.6 (±4.7)	60.4 (±5.7)	45.0 (±3.9)
0.25	**93.4** (±1.9)	**89.8** (±5.3)	**79.7** (±8.0)	**85.5** (±8.5)	**94.9** (±3.3)
0.5	92.5 (±1.4)	88.5 (±4.4)	78.2 (±8.1)	85.2 (±7.1)	94.3 (±2.6)
0.75	91.7 (±2.1)	88.2 (±4.3)	79.0 (±6.8)	82.8 (±6.2)	88.2 (±2.8)
1	92.4 (±2.1)	88.1 (±7.7)	78.9 (±7.3)	83.5 (±6.1)	86.8 (±3.1)

Table S4. Gesture classification accuracy (%) across DB1 to DB5 for different values of λ under 5-shot setting, evaluating the effect of loss balancing. Results (unit: %) are averaged over the first five subjects from each database.

Distance metric	DB1	DB2	DB3	DB4	DB5
Cosine Similarity	87.6 (±3.7)	88.2 (±5.2)	78.1 (±7.4)	81.4 (±5.6)	87.7 (±3.9)
Euclidean Distance	**88.9** (±3.1)	**89.8** (±5.1)	**79.6** (±6.9)	**84.6** (±5.4)	**89.5** (±3.4)
Mahalanobis Distance	53.2 (±5.1)	47.8 (±7.2)	47.8 (±7.6)	55.9 (±10.3)	55.0 (±4.5)
KL Divergence	88.5 (±3.8)	87.6 (±5.0)	79.1 (±5.2)	79.6 (±4.6)	88.4 (±5.5)

Table S5. Cross-session test accuracy (unit: %) across all subjects on the Ninapro DB1–DB5 datasets under a 5-way 5-shot setting.

	DB1	DB2	DB3	DB4	DB5
**Development machine**					
Mean latency (ms)	14.686	14.594	14.967	15.124	14.576
Median latency (ms)	14.474	14.246	14.478	14.392	14.359
Std latency (ms)	1.575	1.123	1.874	2.534	0.687
Peak memory (MB)	0.14	0.55	0.59	0.53	0.45
Params	549,831	615,367	615,367	615,367	746,439
Model size (KB)	2234.51	2490.51	2490.51	2490.51	3002.51
FLOPs (est.)	1,315,640	1,530,940	1,530,940	1,530,940	1,961,540
Throughput (samples/sec)	68.09	68.52	66.81	66.12	68.61
**ARM-device**					
Mean latency (ms)	7.410	7.503	7.636	7.124	7.618
Median latency (ms)	6.881	6.933	7.220	6.783	7.132
Std latency (ms)	2.738	1.983	2.917	1.247	2.511
Peak memory (MB)	0.03	0.04	0.04	0.03	0.03
Params	549,831	615,367	615,367	615,367	746,439
Model size (KB)	2250.31	2506.31	2506.31	2506.31	3018.33
FLOPs (est.)	1,315,640	1,530,940	1,530,940	1,530,940	1,961,540
Throughput (samples/sec)	134.95	133.28	130.96	140.36	131.27

Table S6. Inference benchmarking results for real-time evaluation. Latency, memory usage, and throughput were measured over 500 inference runs (including 50 warm-up runs) with batch size = 1. Experiments were conducted on a development workstation (Intel Core i9-12900KS CPU with 32 GB RAM and an NVIDIA RTX 3070Ti GPU with 8 GB VRAM) and an ARM-based device (Apple M2 CPU, 8-core, 8 GB RAM, macOS 15.6, Python v3.13.0). The results demonstrate low latency and high throughput, supporting the feasibility of real-time EMG decoding across platforms.

Supplementary Figures

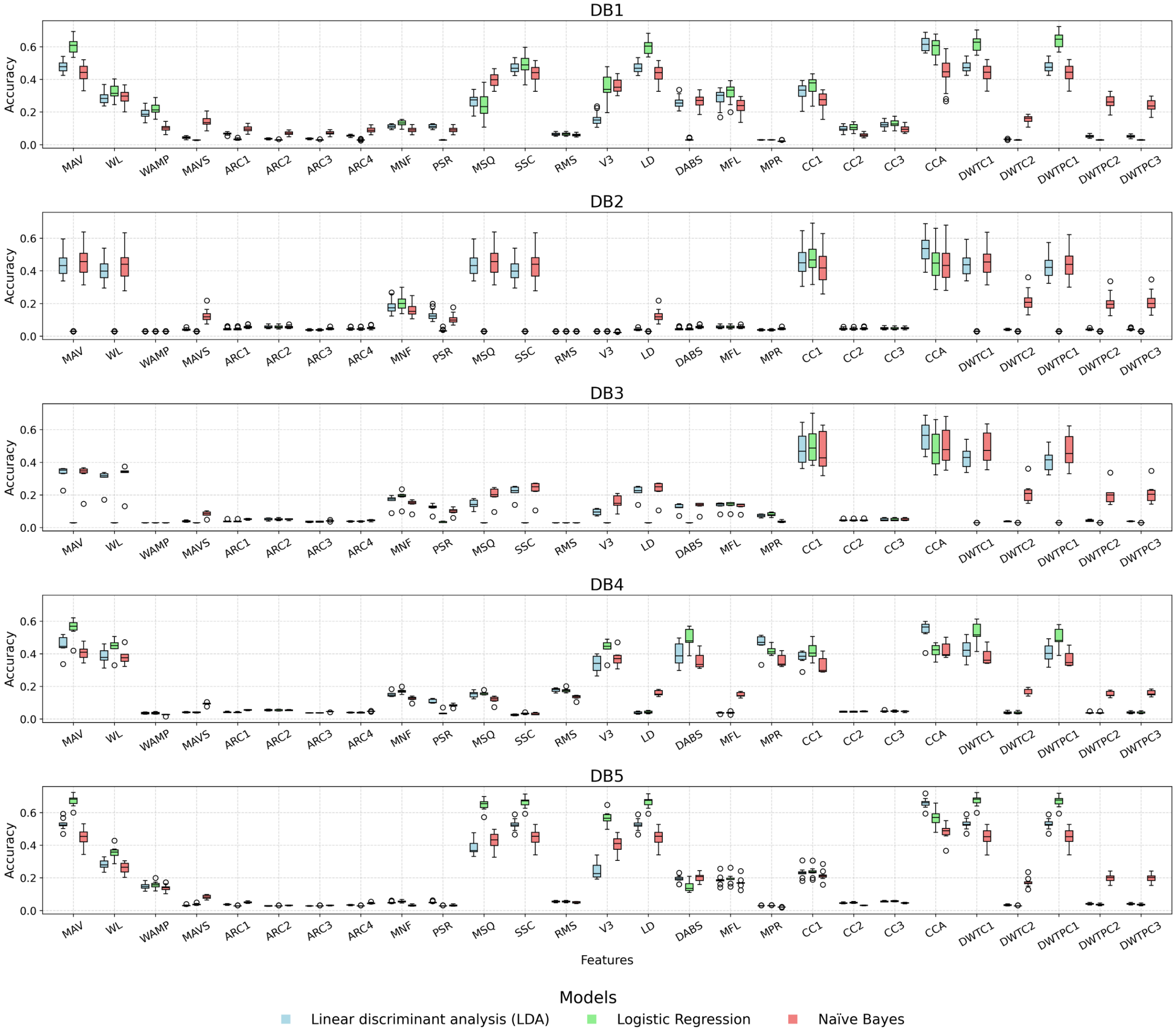

Figure S2. Performance comparison of three simple classifiers (LDA, Logistic Regression, and Naive Bayes) evaluated on inter-session data across five databases. Results are averaged over all subjects and reported for individual univariate feature types. The analysis demonstrates that the CCA feature set enables stronger performance than others, suggesting its inherent capability to extract discriminative and generalizable representations across different subjects, sessions, and datasets.

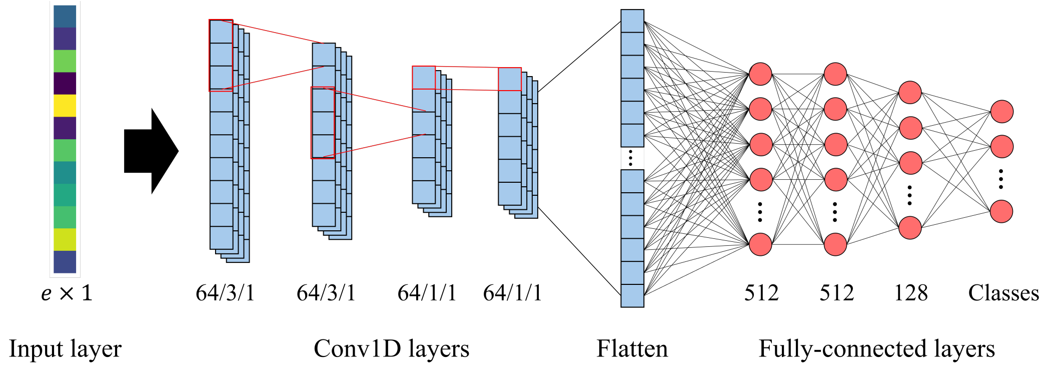

Figure S3. Architecture of the 1D-CNN model. “BN" denotes batch normalization. For the Conv1D layers, the numbers indicate the filter size, kernel size, and stride, respectively (e.g., 64/3/1). For the fully connected layers, the numbers represent the layer dimensions (512 and 128).

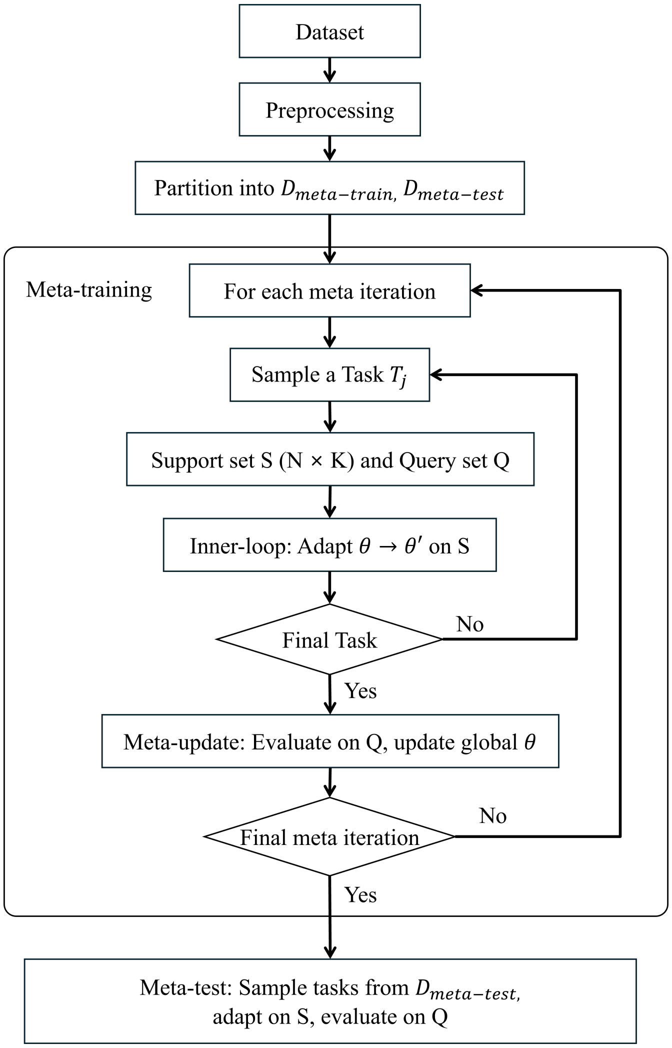

Figure S4. Overview of the few-shot meta-learning procedure with inner-loop adaptation and outer-loop meta-update.

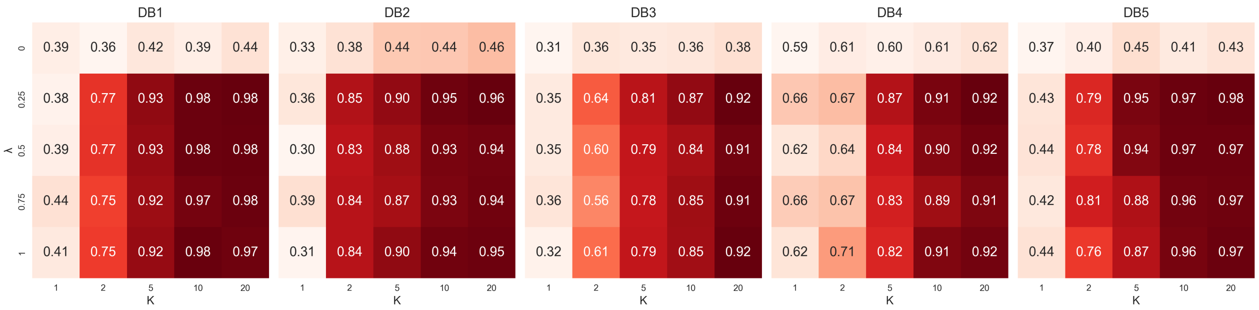

Figure S5. Sensitivity analysis of K-shot and λ across databases. Results are presented as heatmaps under the inter-session setting, averaged over all subjects. Note that each value indicates accuracy.

References

1. Bogert, B. P. The quefrency alanysis of time series for echoes: Cepstrum, pseudoautocovariance, cross-cepstrum and saphe cracking. In *Proc. Symposium Time Series Analysis, 1963*, 209–243 (1963).

2. Oppenheim, A. V. & Schafer, R. W. From frequency to quefrency: A history of the cepstrum. *IEEE signal processing Mag.* **21**, 95–106 (2004).

3. Moawad, A. *et al.* New framework for human activity recognition for wearable gait rehabilitation systems. *Appl. Syst. Innov.* **8**, 53 (2025).

4. Biagetti, G., Crippa, P., Orcioni, S. & Turchetti, C. Homomorphic deconvolution for muap estimation from surface emg signals. *IEEE journal biomedical health informatics* **21**, 328–338 (2016).
